# High mitochondrial sequence divergence in synanthropic flea species (Insecta: Siphonaptera) from Europe and the Mediterranean

**DOI:** 10.1186/s13071-018-2798-4

**Published:** 2018-04-02

**Authors:** Sándor Hornok, Relja Beck, Róbert Farkas, Andrea Grima, Domenico Otranto, Jenő Kontschán, Nóra Takács, Gábor Horváth, Krisztina Szőke, Sándor Szekeres, Gábor Majoros, Alexandra Juhász, Harold Salant, Regina Hofmann-Lehmann, Michal Stanko, Gad Baneth

**Affiliations:** 1 Department of Parasitology and Zoology, University of Veterinary Medicine, Budapest, Hungary; 2 Department for Bacteriology and Parasitology, Croatian Veterinary Institute, Zagreb, Croatia; 3 Department of Veterinary Medicine, University of Bari, Bari, Italy; 4 Plant Protection Institute, Centre for Agricultural Research, Hungarian Academy of Sciences, Budapest, Hungary; 5 Veterinary Authority, Csurgó, Hungary; 6 Koret School of Veterinary Medicine, Hebrew University, Rehovot, Israel; 7 Clinical Laboratory and Center for Clinical Studies, Vetsuisse Faculty, University of Zurich, Zurich, Switzerland; 8 Institute of Parasitology, Slovak Academy of Sciences, Košice, Slovak Republic

## Abstract

**Background:**

Adult fleas are haematophagous ectoparasites of warm-blooded vertebrates, particularly mammals. Among them, the cat flea (*Ctenocephalides felis*) and the human flea (*Pulex irritans*) have high veterinary-medical significance, owing to their cosmopolitan distribution and role in the transmission of important vector-borne pathogens. While the taxonomy of *Ct. felis* has been investigated on a morphological basis during the past decades, its molecular-phylogenetic analyses have been only recently conducted. This study expands the knowledge on *Ct. felis* from hitherto less studied geographical regions, and includes representatives from additional flea families, less investigated with molecular approaches.

**Methods:**

Fleas were collected in four countries of the Mediterranean Basin (Croatia, Italy, Malta and Israel), as well as in Hungary, from domestic and wild carnivores, rodents and humans. The DNA extracts of representative fleas (*n* = 148), belonging to ten species of eight genera, were used for PCR amplification of part of their cytochrome *c* oxidase subunits 1, 2 (*cox*1, *cox*2) and *18S* rRNA genes, followed by sequencing and phylogenetic analyses.

**Results:**

The majority (65.6%) of *Ct. felis felis cox*2 sequences showed 99.4–100% similarity to each other (haplogroup A), whereas those from Malta and Israel had 98.1–98.7% sequence similarity (haplogroup B), and a third sequence from Israel (haplotype C) had as low as 96.3% sequence similarity in comparison with a reference sequence from group “A”. Except for the shape of the head, no consistent morphological differences (e.g. in chaetotaxy) were found between haplogroups “A” and “C”. Haplotypes of *Ct. canis* were genetically more homogenous, with 99.6–100% sequence similarity to each other. However, when *P. irritans* collected from humans was compared to those from three species of wild carnivores, these only had 96.6% *cox*2 similarity. The mouse flea, *Leptopsylla segnis* and the northern rat flea, *Nosopsyllus fasciatus* were both shown to have haplotypes with low intraspecific *cox*2 similarities (96.2 and 94.4%, respectively). Taken together, differences between mitochondrial lineages within four flea species exceeded that observed between two *Chaetopsylla* spp. (which had 97.3% *cox*2 similarity). The topologies of *cox*1 and *cox*2 phylogenetic trees were in line with relevant sequence comparisons. Conversely, *18S* rRNA gene analyses only resolved differences above the species level.

**Conclusions:**

*Ctenocephalides felis felis*, *P. irritans*, *L. segnis* and *N. fasciatus* were shown to have such a high level of mitochondrial gene heterogeneity, that the uniformity of these flea taxa should be reconsidered. Although the present results are limited (especially in the case of *L. segnis* and *N. fasciatus*), there appears to be no geographical or host restriction, which could explain the divergence of these genetic lineages.

**Electronic supplementary material:**

The online version of this article (10.1186/s13071-018-2798-4) contains supplementary material, which is available to authorized users.

## Background

Fleas (Order Siphonaptera) include more than 2500 species of small, wingless insects, which, in the adult stage, are haematophagous ectoparasites of warm-blooded vertebrates [1]. The majority (approximately 95%) of flea species infest mammals [2]. While there are flea families, which associate with a particular mammalian host group (e.g. Vermipsyllidae with carnivores, Ischnopsyllidae with bats), in general, fleas are not strictly host species-specific [1]. Therefore, taxonomically and/ or ecologically related hosts might share flea species. This increases the epidemiological significance of fleas, because they may transmit vector-borne pathogens not only between individuals of the same host species but also between different host species [1].

Although only the minority of flea species are regarded as synanthropic [1], the geographically most widespread and economically most important ones are associated either with humans or with pet animals and rodents concomitant with human presence. The cat flea (*Ctenocephalides felis*) and the human flea (*Pulex irritans*) exemplify such cosmopolitan species, with consequently high veterinary-medical significance [1, 3]. These two flea species can also be characterized by a relatively broad host range, including humans, a variety of carnivores, rodents and ungulates [1, 4].

The taxonomy of *Ct. felis* is in a state of transition. For instance, based on morphological [5] and molecular data [3, 6], it was proposed that two of its subspecies (*Ct. felis orientis*, *Ct. felis damarensis*) should be raised to the rank of species. Recent studies on the phylogeny of *Ct. felis* revealed the existence of formerly unrecognized phylogenetic clades [3, 6].

However, in the latter studies, certain regions of the Globe, in particular, the southern part of central Europe and the Mediterranean Basin were not represented. Also, to the best of the authors' knowledge, no similar molecular phylogenetic studies have been published, which included simultaneously *Ctenocephalides* spp. and *P. irritans*, as well as representatives of the Leptopsyllidae, Vermipsyllidae and Ceratophyllidae. Therefore, the present study was initiated with a primary focus on the phylogenetic analysis of fleas from domestic carnivores (*Ct. felis* and *Ct. canis*) collected in Hungary, Croatia, Italy, Malta and Israel, but also aimed at investigating the human flea (*P. irritans*) and further flea species from rodents and wild carnivores in the same context. Two mitochondrial genes (cytochrome *c* oxidase subunits 1 and 2, i.e. *cox*1, *cox*2) were selected as the main targets of molecular and phylogenetic analyses because these are suitable to study the intra- and interspecific genetic diversity of fleas [3]. Additionally, partial fragment of the *18S* rRNA gene was also amplified, to include a nuclear ribosomal marker for comparison [7].

## Methods

### Sample collection and identification

Fleas were collected from domestic and wild carnivores, rodents, humans and off-host (from the environment) in five countries, in 2014–2017 (Additional file 1). Samples were stored in 96% ethanol. The species of fleas were identified according to morphological keys and descriptions [8–10]. In addition, 14 male and 24 female *Ct. felis felis* specimens from Israel were morphometrically studied according to their haplotypes, determined with *cox*2 PCR and sequencing. Representative specimens of flea species collected from carnivores and rodents (i.e. *P. irritans*, *Archaeopsylla erinacei erinacei*, *Chaetopsylla* spp., *Leptopsylla segnis* and *Ceratophyllus sciurorum*) were also mounted on slides in Canada balsam, after clarification in 10% KOH and increasing concentrations of ethanol for dehydration [3]. Pictures were made with a VHX-5000 digital microscope (Keyence Co., Osaka, Japan).

### DNA extraction

DNA was extracted from individual fleas with the QIAamp DNA Mini Kit (Qiagen, Hilden, Germany) according to the manufacturer’s instruction, including overnight digestion in tissue lysis buffer with 6.6% Proteinase K at 56 °C. The whole body was used for DNA extraction in 124 of the 162 processed fleas. To perform morphological examination of a subset of *Ct. felis felis* specimens (*n* = 38) according to their haplotypes, the DNA was extracted from one hind leg of 24 females, which were large enough to allow manual mincing of one leg, thus leaving other parts intact. In addition, DNA was extracted from 14 males after making an incision dorsally behind the 2nd abdominal tergite, and retaining the exoskeleton after incubation in tissue lysis buffer [3]. Sequence and phylogenetic comparisons between flea species were based on the molecular analysis of 148 DNA samples, obtained from the whole body of 124 fleas and the leg of 24 *Ct. felis felis* females.

### Molecular and phylogenetic analyses

The *cox*2 gene was chosen as the primary target of molecular analyses, because it is suitable to identify and to compare flea species, and it has advantages over *cox*1. These include: (i) the relevant *cox*2 fragment (available in GenBank for several species) is longer than that of *cox*1, plus (ii) the *cox*2 PCR has a high rate of success with one pair of primers, as contrasted to the *cox*1 PCR with mixed success and the necessity to apply further primers [3]. The *cox*2 PCR used here amplifies an approximately 780 bp long fragment of the gene with primers F-Leu (5'-TCT AAT ATG GCA GAT TAG TGC-3') and R-Lys (5'-GAG ACC AGT ACT TGC TTT CAG TCA TC-3') [11]. The reaction was carried out in a final volume of 25 μl, containing 1 U (0.2 μl ) HotStar*Taq* Plus DNA polymerase, 2.5 μl 10× CoralLoad Reaction buffer (including 15 mM MgCl_2_), 0.5 μl PCR nucleotide Mix (0.2 mM each), 0.5 μl (1.0 μM final concentration) of each primer, 15.8 μl ddH_2_O and 5 μl template DNA. For amplification, an initial denaturation step at 95 °C for 5 min was followed by 40 cycles of denaturation at 94 °C for 40 s, annealing at 53 °C for 1 min and extension at 72 °C for 1 min. Final extension was performed at 72 °C for 7 min.

To complement the results obtained with the *cox*2 gene, selected samples (*n* = 101) were also tested with a PCR targeting another mitochondrial marker, the *cox*1 gene. This method amplifies an approximately 550 bp fragment, with primers Cff-F (5'-AGA ATT AGG TCA ACC AGG A-3') and Cff-R (5'-GAA GGG TCA AAG AAT GAT GT-3') [3]. Five μl of template DNA were added to 20 μl reaction mixture, containing 1 U (0.2 μl) HotStar*Taq* Plus DNA polymerase, 2.5 μl 10× CoralLoad Reaction buffer (including 15 mM MgCl_2_), 0.5 μl PCR nucleotide Mix (0.2 mM each), 0.5 μl (1 μM final concentration) of each primer and 15.8 μl ddH_2_O. An initial denaturation step at 95 °C for 5 min was followed by 40 cycles of denaturation at 95 °C for 40 s, annealing at 50 °C for 1 min and extension at 72 °C for 1 min. Final extension was performed at 72 °C for 7 min.

Samples, for which the basic *cox*1 PCR did not yield a positive result, were re-tested with a modified version of the method. This modified *cox*1 PCR, targeting an approximately 680 bp long fragment of the gene, was carried out with the same reaction conditions as above, but using the LCO1490 forward primer (5'-GGT CAA CAA ATC ATA AAG ATA TTG G-3') [3, 12].

In addition, samples (*n* = 35) representing divergent *Ct. felis felis* and *P. irritans* haplotypes, as well as six further species, were analysed with a PCR amplifying an approximately 980 bp long fragment of the nuclear *18S* rRNA gene, with primers 18S a1.0 (5'-GGT GAA ATT CTT GGA YCG TC-3') (forward) and 18S 9R **(**5'-GAT CCT TCC GCA GGT TCA CCT AC-3') (reverse) [7]. Reaction conditions were the same as in the *cox*1 PCR, except for annealing at 55 °C for 1 min.

PCR products were visualized in 1.5% agarose gel. Purification and sequencing were done by Biomi Inc. (Gödöllő, Hungary). Obtained sequences were manually edited, then aligned and compared to reference GenBank sequences (which were selected on the basis of high coverage to sequences from this study) by the nucleotide BLASTN program (https://blast.ncbi.nlm.nih.gov). Representative sequences were submitted to GenBank (see accession numbers in Additional file 1). The MEGA model selection method was applied to choose the appropriate model for phylogenetic analyses, with 1000 resamplings to generate bootstrap values. Phylogenetic analyses were conducted with the Maximum Likelihood method by using MEGA version 6.0 [13], only including GenBank entries with high coverage in comparison with sequences of the present study.

### Statistical analysis

Host-associations of flea species were compared with Fisher’s exact test. Differences were regarded significant when *P* < 0.05.

## Results

### Host associations of flea species

The majority of fleas used for comparative molecular analyses (i.e. 97 out of 148) were identified as *Ct. felis felis*, followed by *Ct. canis* (*n* = 15). *Ctenocephalides felis felis* was significantly more common on cats than on dogs (79 *vs* 17 specimens, discounting one off-host sample), whereas *Ct. canis* occurred significantly more frequently on dogs (*n* = 13) than on cats (*n* = 2) (*P* < 0.0001). This also implies that more *Ct. felis* (*n* = 17) than *Ct. canis* (*n* = 13) specimens were collected from dogs. Two specimens of *Nosopsyllus fasciatus* and one *A. erinacei erinacei* were collected from cats in Italy and Hungary, respectively. *Pulex irritans* (*n* = 11) was only found on humans and wild carnivores (Additional file 1). *Chaetopsylla globiceps* (*n* = 3) and *Ch. trichosa* (*n* = 3) occurred only on fox. Rodents were infested with their “specific” fleas, i.e. rats with *Xenopsylla cheopis* (*n* = 10), mice with *L. segnis* (*n* = 2) and a squirrel with *Ce. sciurorum* (*n* = 3) (Additional file 1). One *N. fasciatus* specimen was also found in the environment.

### Molecular and phylogenetic analyses of the *cox*2 and *cox*1 genes

**Fig. 1:**
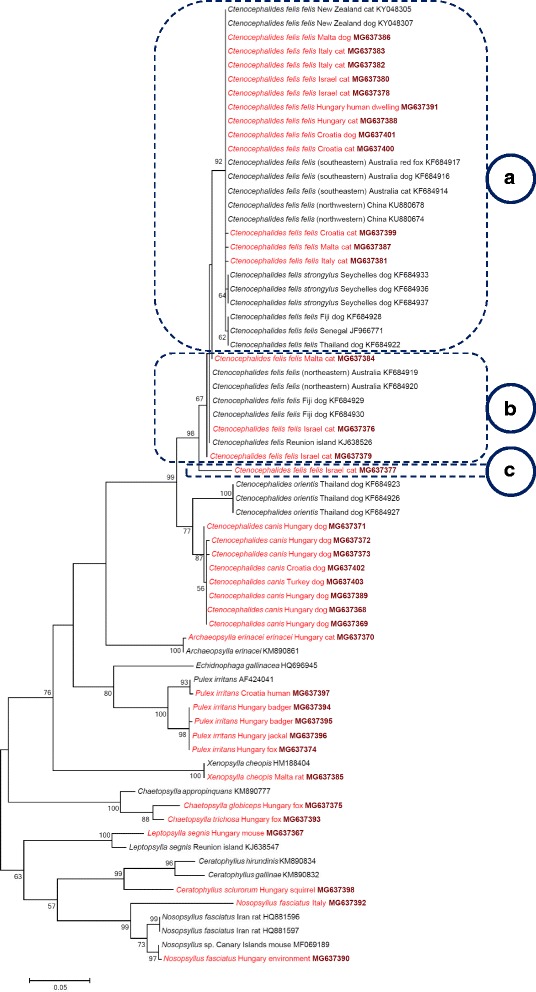
Phylogenetic tree of flea species based on the *cox*2 gene, obtained with the Tamura-Nei model. Sequences obtained in this study are indicated with red colour and bold GenBank accession numbers. After species names, the country and host of origin are shown, if known. A dashed line surrounds three phylogenetic clusters of *Ctenocephalides felis*, labelled with encircled capital letters (**a** to **c**). Branch lengths represent the number of substitutions per site inferred according to the scale shown

**Fig. 2:**
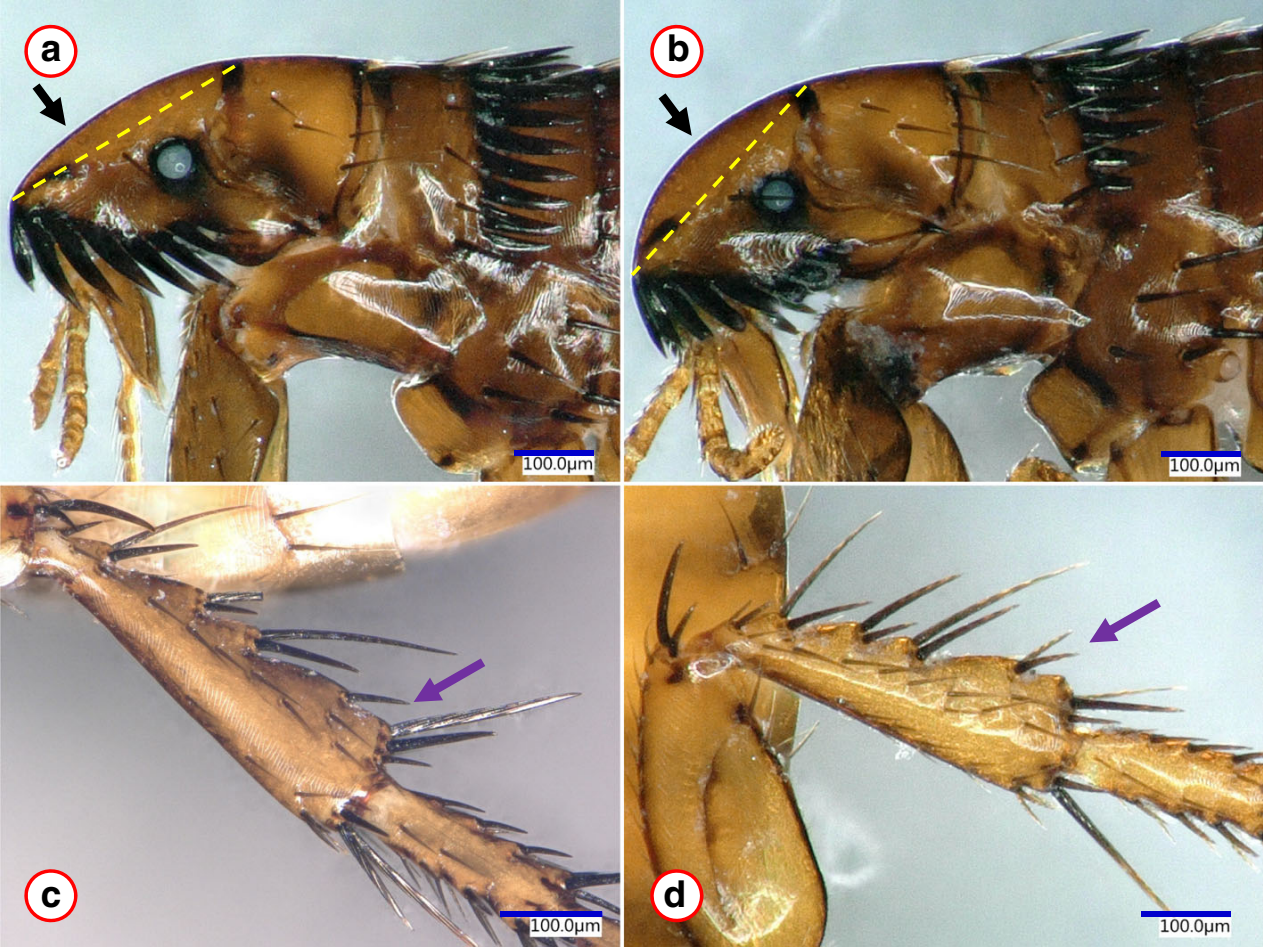
Morphological comparison of *Ctenocephalides felis felis* females representing the most divergent haplotypes. Pictures (**a**) and (**b**) show the head and pronotum from clusters A and C, respectively. The yellow dashed line between the basis of the anterior and dorsal incrassations serves to highlight the more (**a**) or less (**b**) flattened upper front part of the head. **c**, **d** show the hind tibia from clusters A and C, respectively. The purple arrow indicates difference in chaetotaxy, which was, however, found only in one out of four specimens

One hundred and forty-one samples yielded sequencable amplicons in the *cox*2 analyses, and 101 samples were used in the *cox*1 analyses. The latter also included seven DNA extracts, i.e. one *Ct. felis* from Italy, four *X. cheopis* from Malta and two *Ce. sciurorum* from Hungary, for which the *cox*2 gene sequencing was not successful (Additional file 1). The majority of *Ct. felis felis* specimens showed only 0–5 nucleotide differences, i.e. 99.4–100% similarity to reference sequences (Additional file 1) in the amplified part of their *cox*2 and *cox*1 genes (haplogroup A). However, three other haplotypes were more divergent: one haplotype (*cox*2: MG637384, *cox*1: MG668603) collected in Malta, with 9 *cox*2 and 14 *cox*1 nucleotide differences (98.7% and 97.1% sequence similarities, respectively), and two further haplotypes (*cox*2: MG637376, MG637379; *cox*1: MG668605, MG668608) collected in Israel, with 12–13 *cox*2 and 13–14 *cox*1 nucleotide differences (98.1–98.3% and 97.1–97.3% similarities, respectively) compared to the reference sequence (Additional file 1; haplogroup B). Furthermore, one haplotype (*cox*2: MG637377, *cox*1: MG668606), collected exclusively in Israel, was even more divergent, having 26 nucleotide differences (*cox*2: 96.3%, *cox*1: 94.6% sequence similarity) in comparison with the reference sequence (Additional file 1). This divergent haplotype (“C”) was also different from *Ct. damarensis* (*cox*2 gene: 94% similarity with KM890776; *cox*1 gene: 92.8% similarity with KM890909). Phylogenetically, this divergent haplotype clustered separately from all other *Ct. felis felis* isolates (Figs. 1, 4: haplotype C *vs* clusters A, B), supported by moderate to high (*cox*1: 85%, *cox*2: 98%) bootstrap values. In the *cox*2 phylogenetic tree, this bootstrap support was higher than those between *Ct. canis* and *Ct. orientis* (77%) or between *Ch. globiceps* and *Ch. trichosa* (88%) (Fig. 1). On the other hand, females belonging to this highly different haplotype did not show consistent (only occasional) differences of chaetotaxy in comparison with other haplotypes of *Ct. felis felis* (Fig. 2c, d). Nevertheless, the anterodorsal surface of the head was more curved in this category (Fig. 2a, b). Taken together, the above three haplotype clusters (A-C) of *Ct. felis felis* did not show particular host association or any apparent geographical pattern (e.g. all occurred on cats in Israel).

Compared to *Ct. felis felis*, evaluated specimens of *Ct. canis* were genetically more homogenous, i.e. they had only 0–3 *cox*2 and 1–2 *cox*1 nucleotide differences compared to reference sequences, amounting to 99.6–100% sequence similarity (Additional file 1). Phylogenetically, all evaluated *Ct. canis* isolates clustered together, as a sister group to *Ct. orientis* (Figs. 1, 4).

**Fig. 3:**
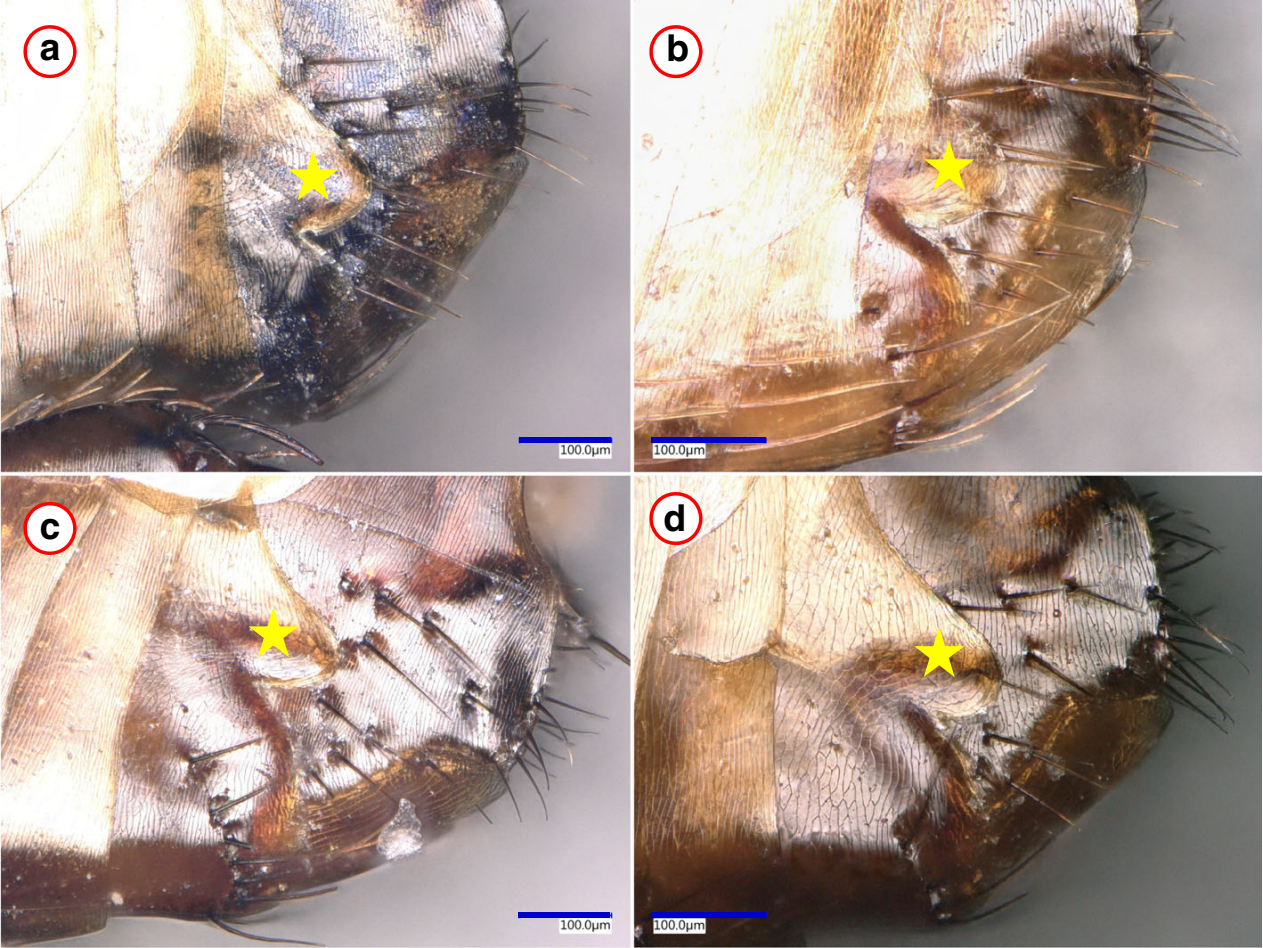
Example of morphological similarities between *Pulex irritans* females from four host species. **a** The 7th sternum of a specimen collected from a human. **b**-**d** The 7th sternum of fleas from wild carnivores (red fox, Eurasian badger and golden jackal, respectively), which belonged to another phylogenetic group. The star marks the hind edge (protrusion and incision) of the 7th sternum, which is a key feature in the identification of *P. irritans*, but did not show differences between the phylogroups

**Fig. 4:**
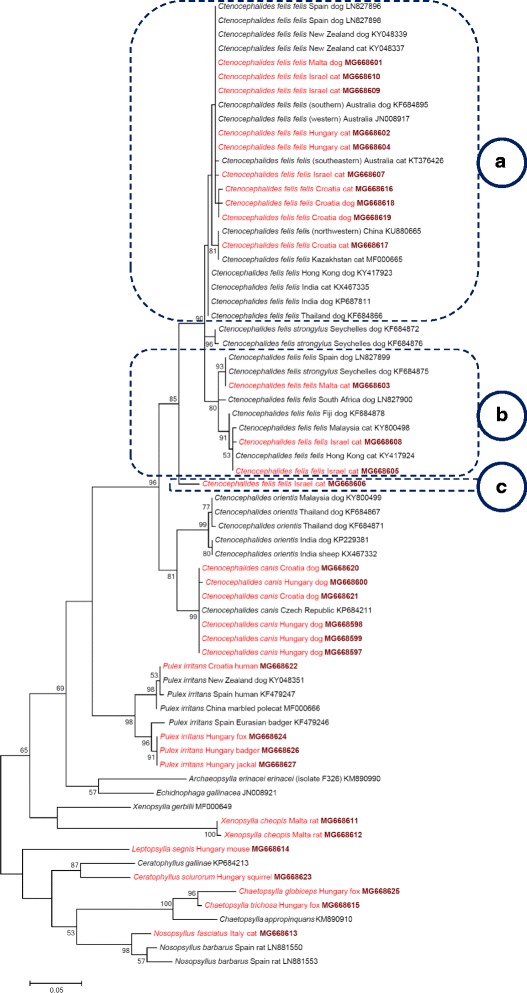
Phylogenetic tree of flea species based on the *cox*1 gene, obtained with the Tamura-3 model. Sequences obtained in this study are indicated with red colour and bold GenBank accession numbers. After species names, the country and host of origin are shown, if known. A dashed line surrounds three phylogenetic clusters of *Ctenocephalides felis*, labelled with encircled capital letters (**a** to **c**). Branch lengths represent the number of substitutions per site inferred according to the scale shown

*Pulex irritans*, collected from humans, had only two nucleotide differences (*cox*2: 99.7%, *cox*1: 99.6% similarity) in comparison with the reference sequences (Additional file 1). In contrast to this, *P. irritans* collected from three species of carnivores had 21 *cox*2 and 19 *cox*1 nucleotide differences (i.e. 96.6% *cox*2 and 96.1% *cox*1 similarity) in comparison with the reference sequences (Additional file 1). Relevant haplotypes belonged to two phylogenetic groups, the separation of which was highly (*cox*2: 100%, *cox*1: 98%) supported (Figs. 1, 4). However, no morphological differences were observed between *P. irritans* specimens from humans and carnivores (see example in Fig. 3).

The two *Chaetopsylla* species from red fox (Additional file 1) had 19 *cox*2 and 18 *cox*1 nucleotide differences from each other (i.e. 97.3% *cox*2 and 96.3% *cox*1 sequence similarity). There were no conspecific sequences retrievable from GenBank.

Mouse fleas (*L. segnis*) collected in Hungary (Additional file 1) had as many as 23 *cox*2 nucleotide differences from a conspecific *cox*2 haplotype reported (and published) from the Reunion Island (KJ638547), amounting to 96.2% similarity.

The *N. fasciatus* specimen from Hungary (Additional file 1) was nearly identical with an isolate from the Canary Islands (MF069189), with one *cox*2 nucleotide difference (99.8% similarity). However, this sample from Hungary had 25 *cox*2 nucleotide differences from *N. fasciatus* collected in Italy (Additional file 1) (425/450 bp, 94.4% similarity). The separation of these two was highly supported (99%) on the *cox*2 phylogenetic tree (Fig. 1).

The remaining three flea species had either minor *cox*2 sequence differences from already published conspecific isolates (*A. erinacei erinacei*, *X. cheopis*) or there was no sequence available in GenBank for comparison (*Ce. sciurorum*) (Additional file 1; Figure 1).

In contrast to a single *cox*2 sequence, two *cox*1 haplotypes of *X. cheopis* were identified. *Xenopsylla cheopis* and a further three species (*L. segnis*, *Ce. sciurorum*, *N. fasciatus*) had no congeneric *cox*1 sequence in this study or conspecific *cox*1 sequence deposited in GenBank for comparison (Additional file 1; Figure 4).

### Molecular and phylogenetic analyses of the *18S* rRNA gene

In these analyses, 35 samples were included (Additional file 1), representing all three *cox*1, *cox*2 haplotypes and phylogenetic clusters. All *Ct. felis felis* specimens (*n* = 12) used for the amplification of the *18S* rRNA gene fragment yielded identical (898/898 bp) *18S* rRNA sequences (MG668628–9, MG668634–5, MG668642), which were also 100% identical with those of *Ct. canis* specimens (MG668632, MG668643: Additional file 1). Similarly, all generated *P. irritans 18S* rRNA sequences (*n* = 9) were 100% (891/891 bp) identical with each other (MG668636, MG668640–1, MG668644) and with a reference sequence (AF423915) from GenBank. There was no difference between *X. cheopis* specimens in this study (*n* = 3), nor between them (MG668630) and the reference sequence (EU336038). However, the *L. segnis* sequence had one nucleotide difference (i.e. 880/881 bp, 99.9% similarity) in comparison with a conspecific reference sequence (DQ298442). Unlike in the case of *Ct. felis felis* and *Ct. canis*, the *18S* rRNA gene sequence and phylogenetic analyses were able to demonstrate differences between the two *Chaetopsylla* species used in this study (MG668637 *vs* MG668638: 895/897 bp, 99.8% similarity).

**Fig. 5:**
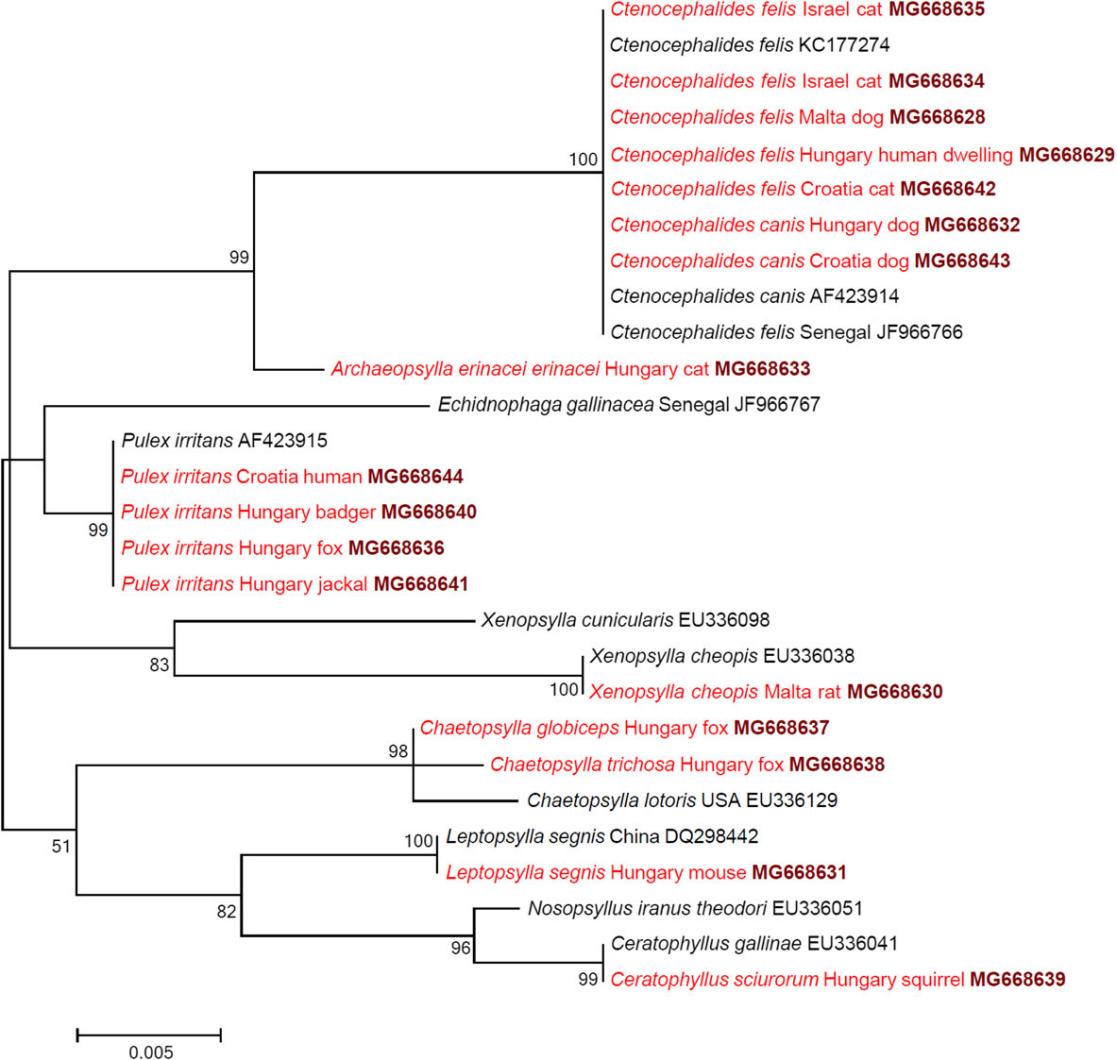
Phylogenetic tree of flea species based on the *18S* rRNA gene, obtained with the K2P model. Sequences obtained in this study are indicated with red colour and bold GenBank accession numbers. After species names, the country and host of origin are shown, if known. Branch lengths represent the number of substitutions per site inferred according to the scale shown

In line with the above sequence comparisons, the *18S* rRNA phylogenetic analysis was only able to resolve differences above the species level (i.e. between families), except for the two *Chaetopsylla* species (Fig. 5). The family-level topology of the *18S* rRNA phylogenetic tree was similar that of the *cox*2 phylogenetic tree (Fig. 1) but was different from the *cox*1 tree (Fig. 4), in which the family Vermipsyllidae (*Ch. globiceps* and *Ch. trichosa*) aligned within the family Ceratophyllidae (genera *Ceratophyllus*, *Nosopsyllus*).

## Discussion

DNA barcoding has become an indispensable tool in the identification and molecular, phylogenetic comparison of animal species [14], including ectoparasites [15]. Here, ten flea species of eight genera were analysed. Three flea species, i.e. *Ct. felis*, *Ct. canis* and *P. irritans* were included with the most representative set of specimens (regarding the individual number and/or a host of origin), thus allowing the assessment of intraspecific genetic diversity. On the other hand, adding seven more species to the analyses in this study (i.e. *A. erinacei*, *X. cheopis*, *Ch. globiceps*, *Ch. trichosa*, *L. segnis*, *Ce. sciurorum*, *N. fasciatus*) strengthened the species/family level resolution and informativeness of phylogenetic trees.

To construct phylogenetic trees informative both on the species and family levels, apart from the fast evolving mitochondrial markers (*cox*1, *cox*2), a conservative nuclear ribosomal gene was also investigated here. It is known that both analysed mitochondrial markers (*cox*1 and *cox*2) can be used effectively to identify species of the genus *Ctenocephalides* (Siphonaptera: Pulicidae) [3]. Although previous data revealed a higher level of nucleotide diversity in the *cox*1 than in the *cox*2 gene [3], this was not consistently seen in the present results. The high mitochondrial diversity of fleas observed here may reflect restricted gene flow, interrelated with the occurrence and movements of flea hosts [16], as outlined below.

Concerning host preferences of the flea species used in the present study, it is well known that *Ct. felis* tends to occur more frequently on dogs than *Ct. canis* [1], as confirmed here. Cats in this study harbored *N. fasciatus* (in Italy) and *A. erinacei erinacei* (in Hungary), both seldom reported from this host species, and usually with low prevalence [17–19]. However, the prevalence of *A. erinacei* on cats may be higher in certain geographical regions, as exemplified by Germany [17]. Among the flea species of wild carnivores, *P. irritans* was collected here from the golden jackal. Based on a recent review [20], the golden jackal was not reported to be a host of *P. irritans* in central Europe. Thus, the present findings contribute to the increasingly recognized epidemiological significance of this wild carnivore, which has an expanding geographical range in Europe [21].

Similarly to previous studies, identical or nearly identical *Ct. felis felis* haplotypes were detected over large geographical distances (e.g. in this study: between Hungary and Israel, or in comparison with other studies: between Hungary, Australia and New Zealand) [3, 22]. This can be explained by the cosmopolitan occurrence and likely transport of this flea species on pet animals, with consequent gene flow between its distant populations. In the present study, different haplotypes of *Ct. felis felis* were also found on the same cat (data not shown), which can be regarded as an indicator of the natural possibility of genetic mixing between haplotypes.

On the other hand, highly divergent *Ct. felis felis* variants, which were associated with the Mediterranean Basin (i.e. cluster B with Malta and Israel, cluster C with Israel), were also identified here. This is in line with previously reported differences in the genetic diversity of *Ct. felis felis* between geographical regions (low in Australia, high in Fiji; [3]).

The mitochondrial haplotype “C”, recognized here for the first time, was clearly separated from the others according to sequence and phylogenetic analyses, suggesting restricted gene flow (possibly also reproductive isolation) between them. In the absence of evident physical (host-related or geographical) barrier between populations representing *Ct. felis felis* haplogroups, the most likely explanation for their divergence is “on host” competition for food resources, which may act as a driver of genetic diversification among parasites [23].

Interestingly, the subspecies *Ct. felis strongylus* formed a uniform clade in *cox*2 but belonged to two different phylogenetic groups in *cox*1 phylogenetic analyses. This is in contrast to previous data [3]. Because the taxonomic status of *Ct. felis strongylus* has long been disputed [3, 5], the present data suggest that it may not be a separate subspecies.

Concerning *Ct. canis*, here a limited number of its specimens were analysed (*n* = 15, from three countries), and this reflected relative mitochondrial DNA homogeneity within this species, which can be interrelated with its less ubiquitous occurrence on pet animals compared to *Ct. felis*. This finding supports a previous study, in which higher numbers of *Ct. canis* samples were collected in two countries, and low *cox*1 genetic variability was demonstrated in comparison with *Ct. felis* [6].

Despite the recognized veterinary-medical importance of *P. irritans*, no reports are available on the molecular-phylogenetic analysis of its specimens associated with different hosts. Here, two deeply diverged mitochondrial lineages of this flea species were shown to exist, i.e. in Hungary and Croatia *P. irritans* associated either with wild carnivores or humans belonged to different *cox*2 haplotype lineages (with high support). However, when examining another mitochondrial marker (*cox*1, for which additional sequences were available in GenBank from other geographical regions), this became less evident, because the “human-associated” haplogroup also contained fleas from domestic and wild carnivores. Therefore, larger scale sampling of *P. irritans* from different hosts in the same location will be necessary to arrive at a conclusion in this context.

This is also the first molecular-phylogenetic analysis including the two *Chaetopsylla* spp., which are the most prevalent representatives of their genus on wild carnivores (especially foxes) in western, central and eastern Europe [24, 25]. These two closely related species remained well separated even in the *18S* rRNA phylogenetic analysis, unlike *Ct. felis* and *Ct. canis*.

The family Leptopsyllidae will deserve future attention in flea phylogeny because this group is heterogenous (containing fleas of birds and small mammals) and was reported to be paraphyletic [26]. Previous studies reported incongruent phylogenetic trees, the one based on the *cox*2 gene placing *Leptopsylla* within Pulicidae [27]. In the present study, phylogenetic analyses of *cox*1 and *cox*2 genes, as well as of the *18S* rRNA gene confirmed the position of *L. segnis* (Leptopsyllidae) as a sister group to the cluster of the Vermipsyllidae and Ceratophyllidae. Importantly, in the latter family, two well-separated phylogenetic lineages of *N. fasciatus* were demonstrated here.

Geographically distant isolates of *Ct. felis felis* (i.e. those from Spain, Malta, Seychelles and South Africa) clustered in the same *cox*1 phylogenetic group (Fig. 4), and dissimilar haplotypes occurred in the same location (Jerusalem, Israel). These findings argue against a geographical pattern in the distribution of relevant haplotypes, i.e. also against the subspecies status of highly divergent mitochondrial lineages demonstrated here. Similarly, no geographical correlation could be demonstrated between clades of the flea species *P. simulans* [16]. In addition, *N. fasciatus* was shown here to be genetically nearly identical over a large geographical distance (i.e. between Hungary and the Canary Islands), but very different between two countries much closer to each other (Hungary and Italy).

The *cox*1 gene is the gold standard for DNA barcoding of species [14]. Based on the similar patterns of intraspecific and interspecific variation in the *cox*1 gene in various animal groups, it was proposed that the threshold for the separation of species should be approximately ten times the intraspecific sequence divergence within the study group [28]. *Ctenocephalides felis* haplotypes in cluster “A” (collected in Malta, Italy, Israel and Hungary) had 0–2 bp differences, as contrasted to the 26 bp difference of haplotype “C” from “A”, thus fulfilling this criterion.

In line with this, sequence and phylogenetic differences between mitochondrial DNA haplogroups within *Ct. felis felis* and *P. irritans* (concerning both *cox*1 and *cox*2 genes), as well as within *L. segnis* and *N. fasciatus* (in the *cox*2 gene) exceeded the level of divergence between closely related species analysed here (i.e. *Ch. globiceps* and *Ch. trichosa*). This was confirmed in phylogenetic analyses. Moreover, this significant (nearly 4%) *cox*2 sequence divergence between *Ct. felis felis* isolates is in the range of sequence divergence between *Ct. felis* and *Ct. canis*.

These findings (also taking into account the absence of consistent morphological differences in chaetotaxy) verify the existence of highly divergent mitochondrial lineages within *Ct. felis*, *P. irritans*, *L. segnis* and *N. fasciatus*. This is the most that can be stated, also considering the difficulties in the morphological delineation of genetic variants among fleas. For instance, chaetotaxy was shown to differ between conspecific individuals or even between two legs of the same flea individual [10]. Similarly, morphological differences between *N. fasciatus* and its morphovariant (as a junior synonym of the same species, *N. barbarus*) did not correspond to molecular genetic differences [29].

In addition, mitochondrial markers may contradict nuclear markers in delineating (cryptic or biological) species, and even substantial differences between mitochondrial lineages (DNA haplogroups) within animal species may not necessarily be supported by nuclear ribosomal markers or by comparison of morphological traits [30]. This is especially relevant when the delineation of cryptic species of arthropods is attempted from mitochondrial DNA [31, 32]. Therefore, to complement the results obtained with two mitochondrial markers, in the present study molecular phylogenetic analysis of the *18S* rRNA gene was also performed with divergent *Ct. felis felis* and *P. irritans* haplotypes, as well as six further species. This nuclear marker could not reproduce the separation of highly divergent mitochondrial lineages within *Ct. felis felis*. On the other hand, while it also failed to demonstrate any differences between the well-established species *Ct. felis* and *Ct. canis* (similarly to what has been reported [33]), this method proved to be suitable to distinguish between three species of the genus *Chaetopsylla* (Fig. 5). Thus, molecular-phylogenetic analyses of the *18S* rRNA gene can still be useful in comparing fleas on the species level (complementing mitochondrial markers) or above [26].

## Conclusions

*Ctenocephalides felis felis*, *P. irritans*, and (with few specimens) *L. segnis* and *N. fasciatus*, were shown to have such a high level of hitherto unknown intraspecific variation (mitochondrial gene heterogeneity), that their taxonomic integrity should be reconsidered. *Ctenocephalides felis* and *P. irritans* have long been recognized as probably the most important flea species of veterinary-medical importance, with special emphasis on their role as blood-sucking arthropods in the transmission of vector-borne pathogens. Therefore, these mitochondrial lineages should be evaluated from the point of view of differences in their competence to transmit disease agents.

## Additional file

Additional file 1: Data of samples used in this study and relevant molecular data. The flea species are ordered vertically according to their taxonomic relationships (i.e. representing Pulicidae, Vermipsyllidae, Leptopsyllidae and Ceratophyllidae). (DOCX 25 kb)

## Abbreviations

*cox*1: cytochrome *c* oxidase subunit 1
*cox*2: cytochrome *c* oxidase subunit 2
