# Supplementary material for: High mitochondrial sequence divergence in synanthropic flea species (Insecta: Siphonaptera) from Europe and the Mediterranean
Source: Parasit Vectors. 2018 Apr 2;11:221. doi: 10.1186/s13071-018-2798-4 (PMC5879554; doi:10.1186/s13071-018-2798-4)
Supplement: Supplementary file 1 — Data of samples used in this study and relevant molecular data. The flea species are ordered vertically according to their taxonomic relationships (i.e. representing Pulicidae, Vermipsyllidae, Leptopsyllidae and Ceratophyllidae). (DOCX 25 kb) [file 13071_2018_2798_MOESM1_ESM.docx]

| **Flea species** | **Country** | **Location** | **Origin (n)** | ***cox2* sequences** | | ***cox1* sequences** | | ***18S* rRNA sequences** | |
| --- | --- | --- | --- | --- | --- | --- | --- | --- | --- |
|  |  |  |  | diff vs ref^6^(n) | acc. numb. | diff vs ref^7^ (n) | acc. numb. | diff vs ref^8^(n) | acc. numb. |
| *Ct. felis* | Malta | Rabat, Zabbar | cat (6), dog (3) | 0 bp vs A (9) | MG637386 | 0 bp vs J (9) | MG668601 | 0 bp vs Q (4) | MG668628 |
|  | Malta | Ta' Xbiex | cat (1) | 9 bp vs A (1) | MG637384 | 14 bp vs J (1) | MG668603 | - | - |
|  | Malta | Paola | cat (1) | 1 bp vs A (1) | MG637387 | 0 bp vs J (1) | MG668601 | - | - |
|  | Italy | Lipari | cat (1) | 2 bp vs A (1) | MG637381 | 0 bp vs J (5) | MG668601 | - | - |
|  | Italy | Lipari | cat (2) | 1 bp vs A (2) | MG637382 |  |  | - | - |
|  | Italy | Lipari | cat (2) | 0 bp vs A (1) | MG637383 |  |  | - | - |
|  | Israel | Jerusalem | cat (22), dog (1) | 12 bp vs A (23) | MG637376 | 13/14 bp (14/1) | MG668605/8 | - | - |
|  | Israel | Jerusalem | cat (7), dog (1) | 26 bp vs A (8) | MG637377 | 26 bp vs J (4) | MG668606 | 0 bp vs Q (2) | MG668634 |
|  | Israel | Jerusalem | cat (7) | 1 bp vs A (7) | MG637378 | 2/1/2 bp (2/1/1) | MG668607/9/10 | 0 bp vs Q (3) | MG668635 |
|  | Israel | Jerusalem | cat (1) | 13 bp vs A (1) | MG637379 | 13 bp vs J (1) | MG668605 |  |  |
|  | Israel | Jerusalem | cat (2) | 0 bp vs A (2) | MG637380 | - | - | - | - |
|  | Hungary | Budapest | cat (1) | 1 bp vs A (2) | MG637388 | 0 bp vs J (1) | MG668602 | 0 bp vs Q (1) | MG668629 |
|  | Hungary | Budapest | human dwelling | 2 bp vs A (1) | MG637391 | 0 bp vs J (1) | MG668604 |  |  |
|  | Croatia | Pula | cat (1) | 2 bp vs A (1) | MG637399 | 2 bp vs J (1) | MG668616 | 0 bp vs Q (1) | MG668642 |
|  | Croatia | various^2^ | cat (8), dog (8) | 1 bp vs A (16) | MG637401 | 1/2 bp vs J (1/3) | MG668618/9 | - | - |
|  | Croatia | various^3^ | cat (16), dog (4) | 4 bp vs A (20) | MG637400 | 5 bp vs J (10) | MG668617 | 0 bp vs Q (1) | MG668642 |
| *Ct. canis* | Hungary | Eger | dog (1) | 2 bp vs B (1) | MG637368 | 1 bp vs K (1) | MG668597 | 0 bp vs R (2) | MG668632 |
|  | Hungary | various^4^ | dog (4), cat (1) | 1 bp vs B (5) | MG637369 | 1/2 bp vs K (2/3) | MG668597/9 |  |  |
|  | Hungary | Jászberény | dog (1) | 2 bp vs B (1) | MG637371 | 1 bp vs K (2) | MG668597 | - | - |
|  | Hungary | Felsőtárkány | dog (1) | 3 bp vs B (1) | MG637372 |  |  | - | - |
|  | Hungary | Debrecen | cat (1) | 2 bp vs B (1) | MG637373 | 2 bp vs K (1) | MG668600 | - | - |
|  | Hungary | Budapest | dog (1) | 0 bp vs B (1) | MG637389 | 2 bp vs K (1) | MG668598 | - | - |
|  | Croatia | various^5^ | dog (4) | 1 bp vs B (4) | MG637402 | 1/2 bp vs K (1/3) | MG668620/1 | 0 bp vs R (2) | MG668643 |
|  | (Turkey)^1^ | unknown | dog (1) | 0 bp vs B (1) | MG637403 | - | - | - | - |
| *A. erinacei* | Hungary | Budapest | cat (1) | 3 bp vs C (1) | MG637370 | - | - | nr. (1) | MG668633 |
| *P. irritans* | Croatia | Brnaze, Drniš | human (2) | 2 bp vs D (5) | MG637397 | 2 bp vs L (5) | MG668622 | 0 bp vs S (4) | MG668644 |
|  | Hungary | Porrog | Eurasian badger (1) | 21 bp vs D (1) | MG637394 | 19 bp vs L (2) | MG668626 | 0 bp vs S (2) | MG668640 |
|  | Hungary | Porrog | Eurasian badger (1) | 21 bp vs D (1) | MG637395 |  |  |  |  |
|  | Hungary | Kaszó | golden jackal (1) | 21 bp vs D (1) | MG637396 | 19 bp vs L (1) | MG668627 | 0 bp vs S (1) | MG668641 |
|  | Hungary | Szigetköz | red fox (1) | 21 bp vs D (3) | MG637374 | 19 bp vs L (2) | MG668624 | 0 bp vs S (2) | MG668636 |
| *X. cheopis* | Malta | Rabat, Zabbar | norway rat (5) | 0 bp vs E (6) | MG637385 | 0/1 bp vs M (8/2) | MG668611/2 | 0 bp vs T (3) | MG668630 |
| *Ch. globiceps* | Hungary | Szigetköz | red fox (1) | 39 bp vs F (3) | MG637375 | 31 bp vs N (2) | MG668625 | 3 bp vs U (2) | MG668637 |
| *Ch. trichosa* | Hungary | Szigetköz | red fox (1) | 31 bp vs F (3) | MG637393 | 21 bp vs N (2) | MG668615 | 5 bp vs U (2) | MG668638 |
| *L. segnis* | Hungary | Tar | house mouse (2) | 23 bp vs G (2) | MG637367 | nr. (2×) | MG668614 | 1 bp vs V (1) | MG668631 |
| *Ce. sciurorum* | Hungary | Budapest | red squirrel (1) | 48 bp vs H (1) | MG637398 | 42 bp vs O (3) | MG668623 | 2 bp vs X (2) | MG668639 |
| *N. fasciatus* | Italy | Lipari | cat (2) | 19 bp vs I (2) | MG637392 | 27 bp vs P (1) | MG668613 | - | - |
|  | Hungary | Budapest | cemetery | 16 bp vs I (1) | MG637390 | - | - | - | - |

**Abbreviations**: sp. = species; ssp. = subspecies; diff = number of nucleotides different in comparison with the reference sequence (see below); ref = reference sequence (see below); acc. numb. = accession number; *Ct.* = *Ctenocephalides*; *A.* = *Archaeopsylla*; *P.* = *Pulex*; *X.* = *Xenopsylla*; *Ch.* = *Chaetopsylla*; *L.* = *Leptopsylla*; *Ce.* = *Ceratophyllus*; *N.* = *Nosopsyllus*; bp = base pair; nr. = no reference sequence

**Countries:** 1 - The flea was removed in Croatia from a dog returning from Turkey, i.e. the most likely country of origin for this flea is Turkey.

**Various locations** (according to superscript): 2 - Vukovar, Pelješac, Buševec, Velika Gorica, Prikraj, Markuševec Turpoljski, Zagreb, Osijek, Gornj Laduč; 3 - Vukovar, Osijek, Bjelovar, Jastrebarsko, Dumovec, Zagreb, Hrvatska dubica, Prikraj, Velika Gorica, Sesvete, Đurđekovec; 4 - Eger, Budapest, Törökbálint; 5 - Zagreb, Buševec, Velika Gorica, Stari Glog

^6^**Reference sequences of *cox*2 gene:** A = KY048307 (*Ct. felis*, coverage: 696 bp); B = MG637389 (*Ct. canis*, coverage 696 bp); C = KM890861 (*Archaeopsylla erinacei erinacei*, coverage 678 bp); D = AF424041 (*Pulex irritans*, coverage 624 bp); E = HM188404 (*Xenopsylla cheopis*, coverage 696 bp); F = KM890777 (*Ch. appropinquans*, coverage 657 and 656 bp respectively); G = KJ638547 (*Leptopsylla segnis*, coverage 607 bp); H = KM890832 (*Ce. gallinae*, coverage 662 bp); I = HQ881596 (*N. fasciatus*, coverage 403 and 631 bp, respectively)

^7^**Reference sequences of *cox*1 gene:** J = KY048337 (*Ct. felis*, coverage: 479 bp); K = KP684211 (*Ct. canis*, coverage 487 bp); L = KY048351 (*Pulex irritans*, coverage 489 bp); M = own Malta Xc18 (*Xenopsylla cheopis*, coverage 489 bp); N = KM890910 (*Ch. appropinquans*, coverage 367 bp); O = KP684213 (*Ce. gallinae*, coverage 489 bp); P = LN881553 (*N. barbarus*, coverage 459 bp)

^8^**Reference sequences of *18S* rRNA gene:** Q = KC177274 (*Ct. felis*, coverage: 898 bp); R = AF423914 (*Ct. canis*, coverage 891 bp); S = AF423915 (*Pulex irritans*, coverage 891 bp); T = EU336038 (*Xenopsylla cheopis*, coverage 899 bp); U = EU336129 (*Ch. lotoris*, coverage 897 bp); V = DQ298442 (*Leptopsylla segnis*, coverage 881 bp); X = EU336041 (*Ce. gallinae*, coverage 891 bp)
